# Short Course of Insulin Treatment versus Metformin in Newly Diagnosed Patients with Type 2 Diabetes

**DOI:** 10.3390/jcm7090235

**Published:** 2018-08-23

**Authors:** Marta Seghieri, Eleni Rebelos, Andrea Mari, Luigi Sciangula, Carlo Giorda, Ele Ferrannini

**Affiliations:** 1 Department of Clinical and Experimental Medicine, University of Pisa, 56126 Pisa, Italy; martaseghieri@hotmail.co.uk (M.S.); nrebel1@live.com (E.R.); 2 CNR Institute of Neuroscience, 35127 Padua, Italy; andrea.mari@cnr.it; 3 ASST Lariana, Diabetes Unit, Ospedale Mariano Comense, 22066 Mariano Comense, Italy; luigi.sciangula@asst-lariana.it; 4 Diabetes & Endocrine Unit, ASL Torino 5, 10123 Chieri, Italy; giordaca@tin.it; 5 CNR Institute of Clinical Physiology, 56126 Pisa, Italy

**Keywords:** type 2 diabetes, diabetes remission, insulin treatment, newly-diagnosed diabetes, metformin

## Abstract

The ß-cell dysfunction of type 2 diabetes is partly reversible. The optimal time window to induce glycemic remission is uncertain; short courses of insulin treatment have been tested as a strategy to induce remission. In a pilot study in 38 newly-diagnosed patients, we assessed the time-course of insulin sensitivity and ß-cell function (by repeat oral glucose tolerance tests) following a 6-week basal insulin treatment compared to metformin monotherapy in equipoised glycemic control. At 6 weeks, insulin secretion and sensitivity were increased in both groups whilst ß-cell glucose sensitivity was unchanged. From this time onwards, in the insulin group glycemia started to rise at 3 months, and was no longer different from baseline at 1 year. The initial improvement in insulin secretion and sensitivity dissipated. In the metformin group, fasting plasma glucose and HbA_1c_ levels reached a nadir at 8 months, at which time insulin secretion, glucose and insulin sensitivity were significantly better than at baseline and higher than in the insulin group. A short course of basal insulin in newly-diagnosed patients does not appear to offer clinical advantage over recommended initiation with metformin.

## 1. Introduction

There is now ample evidence that the ß-cell dysfunction that defines type 2 diabetes can be, at least in part, reversed, leading to clinical remission in an interesting fraction of patients [1]. Whether there exists a unique time window to induce a durable glycemic remission is uncertain. Early diabetes is generally regarded as the phase that optimizes the chances of inducing remission [2], although experience with bariatric surgery has shown that large improvements in glycemic control can be achieved even in non-obese patients with long-standing, insulin-treated type 2 diabetes [3]. Short courses of insulin treatment have been tested as a strategy to induce remission, with variable success (reviewed in [4]). With regard to the mechanisms of insulin-induced diabetes remission, the focus appears to be on the ability of insulin to improve insulin secretion, although in post-bariatric patients insulin resistance also abates as a result of the large weight loss [5].

We were interested in examining the time trajectory of the principal pathogenetic mechanisms—namely, ß-cell function and insulin sensitivity—underlying the glycemic improvement that a short course of insulin can induce in newly-diagnosed patients. As a comparator in the study, we opted for metformin, the standard first-line pharmacological therapy; in addition, we tried to equipoise glycemic control between metformin and insulin during the induction phase in order to give even weight to both treatments. We expected to gain sufficient insight into the mechanistic time-course to guide a larger clinical trial.

## 2. Methods

Thirty-eight subjects with newly diagnosed diabetes were allocated—in a 1:1 ratio—to a short course of insulin treatment or metformin; recruitment was opportunistic. The study was approved by the Ethics Committee and registered with EudraCT (#2011-001509-28); all patients gave their written informed consent before they participated in the study. The study was conducted in accordance with the Declaration of Helsinki. As this was an exploratory pilot trial, it was not powered for a clinical outcome (diabetes remission rate).

Insulin treatment consisted of a daily glargine injection for 6 weeks; in the other arm, metformin was started and continued throughout the follow-up period at tolerated doses. Basal insulin therapy was gently titrated aiming at matching the HbA_1c_ expected from metformin treatment at 6 weeks (i.e., −0.8%) while carefully avoiding hypoglycemia; the final insulin dose averaged 0.12 ± 0.01 UI/kg. The study protocol consisted of performing sequential 75-g oral glucose tolerance tests (OGTT), the first at randomization (baseline), the second at the end of the 6-week insulin treatment period, and the other three at 3, 8, and 12 months thereafter. On the OGTT, blood was sampled at frequent intervals for 3 h for substrate (glucose and free fatty acids (FFA)) and hormone (insulin, glucagon, GLP-1) measurements.

The main parameters of ß-cell function, i.e., ß-cell glucose sensitivity (=mean slope of the insulin secretion/glucose concentration dose-response function) and insulin secretion at a fixed glucose concentration (=parallel shift of the dose-response), were calculated from mathematical modelling of plasma glucose and C-peptide responses to oral glucose, as described [6]; insulin sensitivity was indexed as the OGIS (=oral glucose insulin sensitivity index). Using data from historical non-diabetic control subjects of similar age and body mass index (BMI) (*n* = 53, ß-cell glucose sensitivity = 112 ± 55 pmol·min^−1^·m^−2^·mM^−1^ and OGIS = 398 ± 45 mL·min^−1^·m^−2^, mean ± SD) [7] and an alpha of 0.05, we calculated a power of 85% for both parameters. Statistical analysis was carried out with the use of the Mann Whitney test for group differences and the Wilcoxon signed rank test for paired data.

## 3. Results and Conclusions

The baseline clinical and metabolic characteristics of the 2 groups of patients were well matched (Table 1). Of note is that at baseline, despite their relatively mild presenting hyperglycemia, our patients had already markedly compromised ß-cell glucose sensitivity and insulin sensitivity as compared to non-diabetic subjects (see above-mentioned reference values). At 6 weeks, both fasting and 2-h plasma glucose concentrations and HbA_1c_ were significantly reduced in both groups to a similar extent (Table 2), documenting the successful initial matching of glycemic endpoints. These changes from baseline were associated with an increase in insulin secretion at fixed (9 mM) glucose concentrations (i.e., an upward shift of the dose-response), and an increase in insulin sensitivity, whereas ß-cell glucose sensitivity had not changed significantly in either group.

From this time onwards, the time-course of the two groups differed markedly. In the insulin group, fasting glycemia started to rise already at 3 months, and was no longer significantly different from baseline at 1 year. Two-hour plasma glucose and HbA_1c_ levels lagged behind fasting glucose but showed the same upward trend. Whilst glucose sensitivity remained low, the initial improvement in insulin secretion and insulin sensitivity dissipated with time. In the metformin group, fasting and 2-h plasma glucose and HbA_1c_ levels reached a nadir at 8 months, at which time insulin secretion, glucose sensitivity and insulin sensitivity were significantly better than at baseline and higher than in the insulin group. Over the next 5 months, however, ß-cell glucose sensitivity fell back and, in parallel, all glycemic outcomes deteriorated. There were no significant changes over time in BMI, plasma FFA, glucagon or GLP-1 responses to oral glucose in either treatment group.

The current results confirm that in obese patients with newly discovered type 2 diabetes, ß-cell function is already profoundly impaired despite the modest presenting hyperglycemia; insulin sensitivity also is compromised, though to a lesser extent than ß-cell glucose sensitivity. Within the limits of a small size, the indications from the time trajectory data are that (a) a 6-week course of basal insulin treatment results in a short-lived improvement in glycemic endpoints, mainly due to an equally transient increase in insulin sensitivity and absolute insulin secretion. In contrast, ß-cell glucose sensitivity is not detectably improved; and (b) even the effectiveness of metformin appears to diminish within a year and to call for rescue therapy. The lack of significant changes in markers of lipolysis (i.e., circulating FFA levels) and glucose-modifying hormones (glucagon and GLP-1) provides indirect support for these conclusions.

It is plausible that a tighter glucose control achieved through more intensive insulin treatment—using continuous subcutaneous insulin infusion [8,9] or multiple daily injections [9,10] targeting fasting plasma glucose levels between 4 and 6 mmol/L—would be able to enhance ß-cell function, particularly in patients with better preserved initial ß-cell function and shorter-onset disease [9]. Although rate and duration of remission were not endpoints in our limited database, a HbA_1c_ ≤ 6.2% at 3 months was predicted by a lower baseline HbA_1c_ (OR = 0.46; 95% CI: 0.16–0.84 for each 0.2% difference in HbA_1c_) regardless of treatment. However, the other independent predictors in multiple logistic analysis were a higher baseline insulin sensitivity (OR = 1.77; 95% CI: 1.13–3.84 for each 10 units) and a lower age at diagnosis (OR = 0.43; 95% CI: 0.12–0.96 for each 5 years). While the impact of age and baseline HbA_1c_ was expected, that insulin sensitivity would supersede ß-cell glucose sensitivity is at variance with previous estimates [8,9,10]. Whether this difference is due to methodology, type and duration of insulin treatment or patient phenotype (or combinations thereof) cannot be decided from the current results. What our pilot effort nevertheless suggests is that a short course of basal insulin under ‘real life’ conditions (i.e., carefully avoiding hypoglycemia) in newly-diagnosed, mildly hyperglycemic patients (often disinclined towards injections and insulin) does not appear to offer any clinical advantage over recommended standard-of-care initiation with metformin. The progressive loss of efficacy of metformin monotherapy in our small sample is in line with the findings of larger observational studies [11]. 

## Figures and Tables

**Table 1:** Clinical and metabolic characteristics.

	Insulin (*n* = 19)	Metformin (*n* = 19)	*p*
Sex (M/F)	13/8	13/7	n.s.
Age (years)	61 ± 2	59 ± 2	n.s.
BMI (kg/m^2^)	31.5 ± 1.3	31.6 ± 1.5	n.s.
Fasting glucose (mmol/L)	8.3 ± 0.3	8.0 ± 0.4	n.s.
HbA_1c_ (%)	7.6 ± 0.2	7.5 ± 0.3	n.s.
Fasting insulin (pmol/L)	98 [54]	103 [51]	n.s.
Insulin_AUC_ (nmol·L^−1^·h^−1^)	74.9 [52.9]	103.7 [94.1]	n.s.
FFA_AUC_ (mmol·L^−1^·h^−1^)	56.6 [21.6]	57.3 [19.9]	n.s.
Glucagon_AUC_ (nmol·L^−1^·h^−1^)	5.34 [4.18]	6.91 [5.72]	n.s.
GLP-1_AUC_ (nmol·L^−1^·h^−1^)	10.6 [6.7]	13.1 [4.4]	n.s.

Entries are mean ± SEM or median [interquartile range]; BMI = body mass index; FFA = nonesterified fatty acids; AUC = area-under-concentration curve.

**Table 2:** Time-course of glycaemic outcomes and their physiological determinants.

	Baseline	Week 6	Week 14	Week 38	Week 58
**Fasting Plasma Glucose (mmol/L)**					
Insulin	8.3 ± 0.3	7.0 ± 0.3 *	7.7 ± 0.5 *	7.5 ± 0.3 *	8.2 ± 0.4
Metformin	8.0 ± 0.4	6.8 ± 0.3 *	6.6 ± 0.3 *	6.2 ± 0.3 *^,§^	7.8 ± 1.0
**2-h Plasma Glucose (mmol/L)**					
Insulin	15.4 ± 0.9	13.4 ± 1.1 *	12.8 ± 1.1 *	14.7 ± 1.0	17.3 ± 1.1
Metformin	14.1 ± 1.1	12.6 ± 0.8 *	12.4 ± 1.4 *	10.7 ± 1.2 *^,§^	13.3 ± 1.6 ^§^
**HbA_1c_ (%)**					
Insulin	7.6 ± 0.2	6.9 ± 0.1 *	6.5 ± 0.1 *	6.7 ± 0.2 *	6.7 ± 0.2 *
Metformin	7.5 ± 0.3	6.7 ± 0.3 *	6.3 ± 0.2 *	6.0 ± 0.1 *^,§^	6.6 ± 0.3 *
**Insulin Secretion at 9 mM Glucose (pmol·min^−1^·m^−2^)**					
Insulin	197 [213]	276 [160] *	276 [262] *	260 [220]	201 [176]
Metformin	257 [207]	301 [173] *	322 [243] *	455 [354] *^,§^	242 [181]
**ß-cell Glucose Sensitivity (pmol·min^−1^·m^−2^·mM^−1^)**					
Insulin	39 [32]	36 [21]	40 [45]	40 [38]	37 [56]
Metformin	52 [42]	42 [37]	39 [40]	62 [50] *	45 [39]
**Insulin Sensitivity (mL·min^−1^·m^−2^)**					
Insulin	270 ± 8	307 ± 15 *	292 ± 18	296 ± 10	264 ± 11
Metformin	263 ± 11	300 ± 11 *	294 ± 16	317 ± 18 *	294 ± 21

Entries are mean ± SEM or median [interquartile range]; * *p* ≤ 0.05 vs. baseline; ^§^ *p* ≤ 0.05 vs. insulin.
